# Supplementary material for: Dynamical Buffering of Reconfiguration Dynamics in Intrinsically Disordered Proteins
Source: JACS Au. 2026 Mar 2;6(3):1900–13. doi: 10.1021/jacsau.5c01753 (PMC13014269; doi:10.1021/jacsau.5c01753)
Supplement: Supplementary file 1 [file au5c01753_si_001.pdf]

Supplementary Information for: Dynamical buffering of reconfiguration dynamics in intrinsically disordered proteins

Miloš T. Ivanović,[†] Andrea Holla,[†] Mark F. Nüesch,[†] Valentin von Roten,[†] Benjamin Schuler,[*,†,‡] and Robert B. Best[*,¶]

†*Department of Biochemistry, University of Zurich, Zurich 8057, Switzerland*

‡*Department of Physics, University of Zurich, Zurich 8057, Switzerland*

¶*Laboratory of Chemical Physics, National Institute of Diabetes and Digestive and Kidney Diseases, National Institutes of Health, Bethesda, MD 20892-0520*

E-mail: schuler@bioc.uzh.ch; robert.best2@nih.gov

Supporting Figures

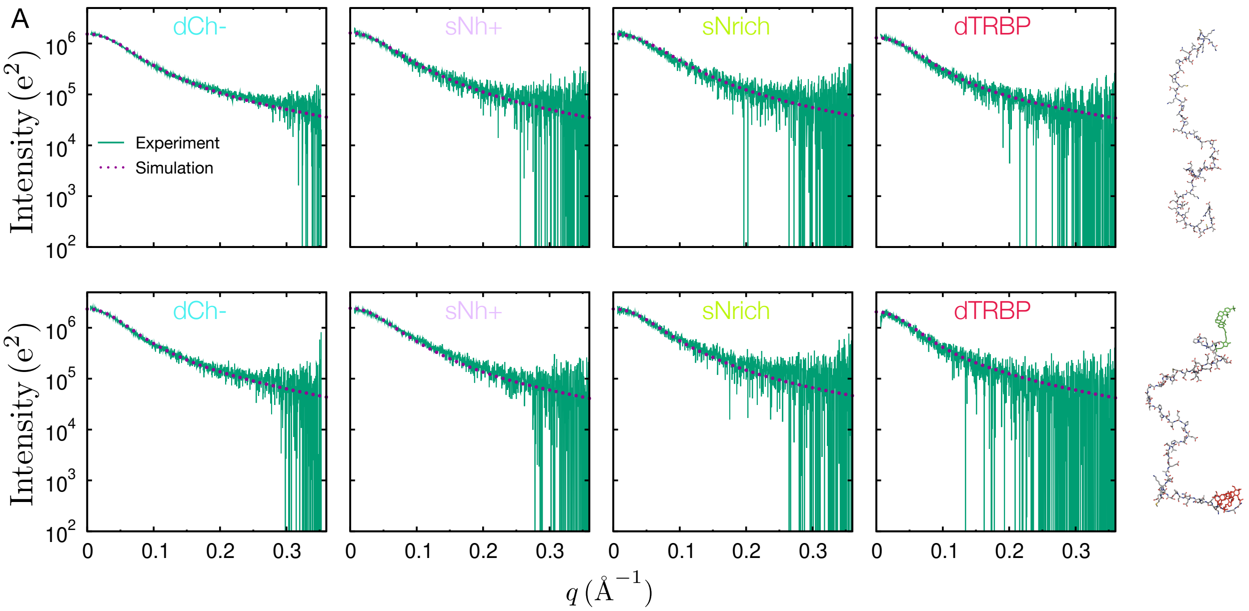

Figure S1: Comparison of the experimental SAXS curves with those calculated from the simulations. (A) without dyes; (B) with dyes (note: in the experiment, IDRs were double-labeled with Alexa 488,[1] while in the simulation, Cy3B + CFR660R were used).

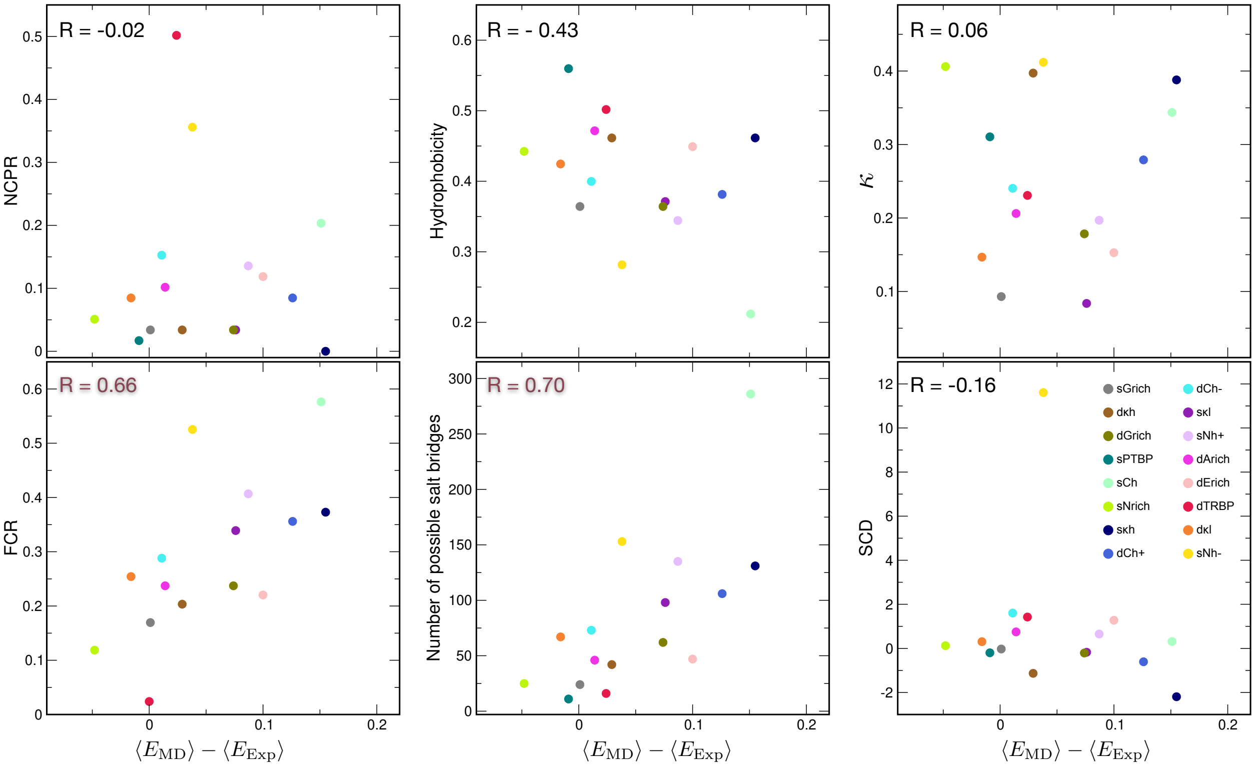

Figure S2: Correlations between the deviation of simulated and experimental FRET efficiencies and net charge per residue (NCPR), fraction of charged residues (FCR), hydrophobicity, number of possible salt bridges, κ parameter[2] and sequence charge decoration (SCD).[3]

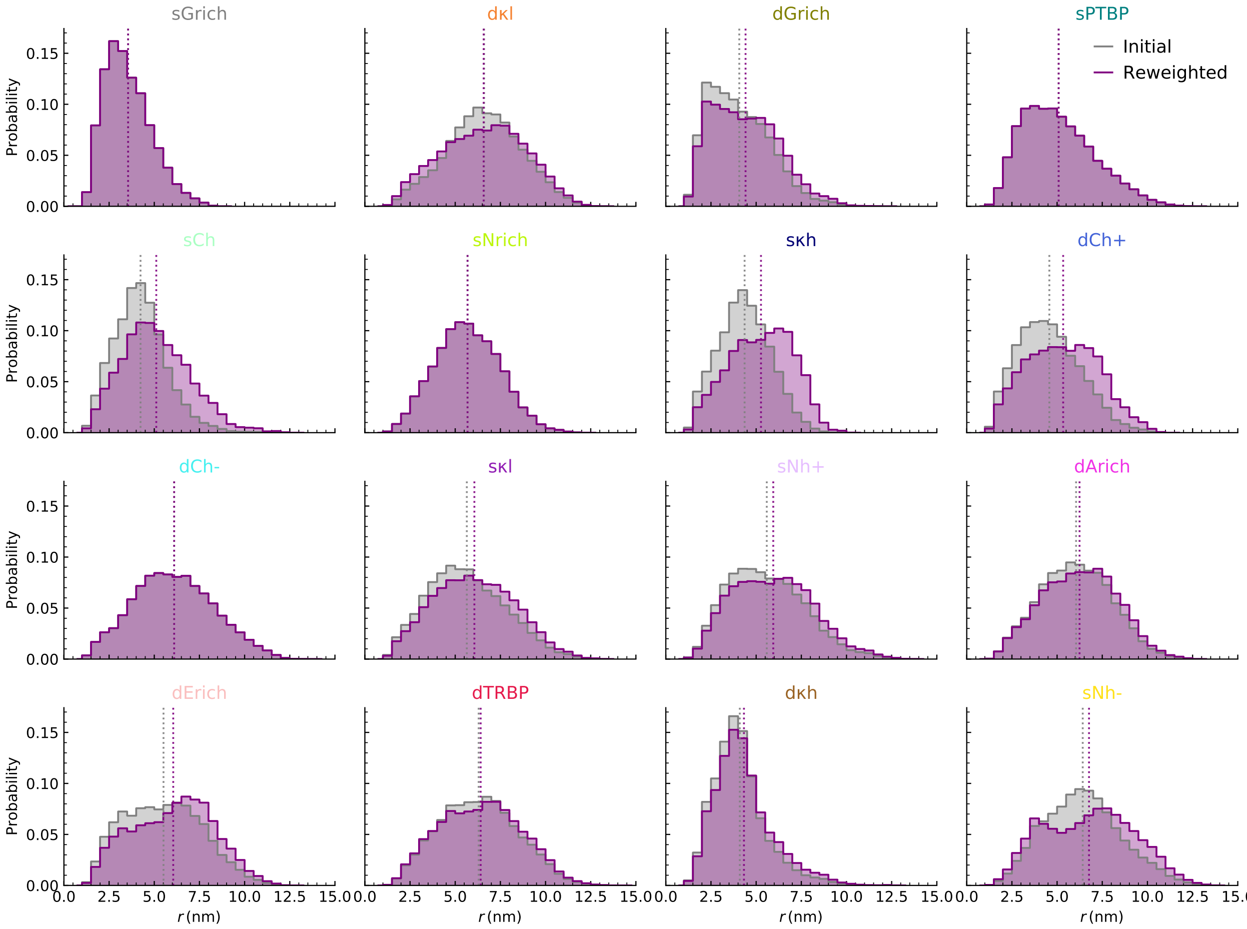

Figure S3: Dye-dye distance distributions before and after reweighting. The vertical lines represent the average values.

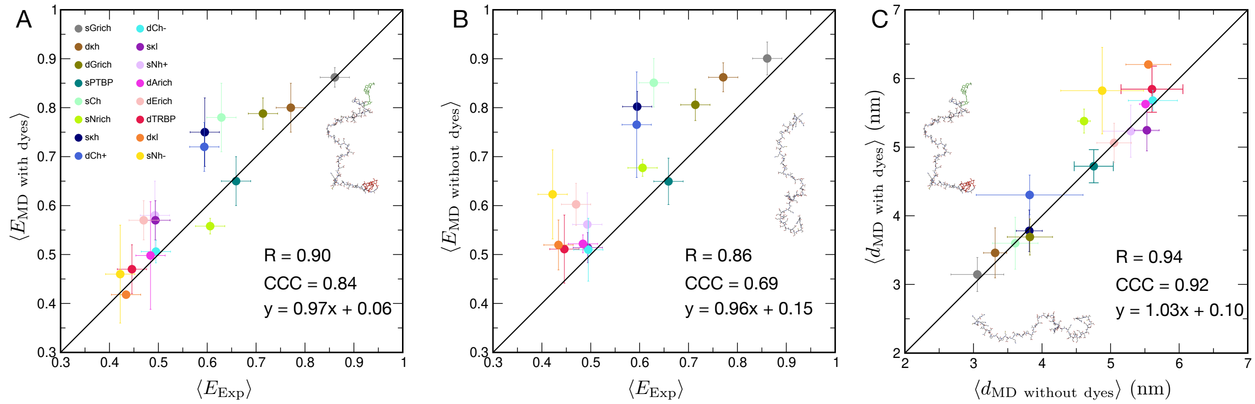

Figure S4: Comparison of experimental transfer efficiencies and transfer efficiencies calculated from simulations with (A) and without (B) dyes. Transfer efficiencies from simulations with dyes are slightly lower than those from simulations without dyes (see also Fig. 4A), suggesting slightly more extended IDRs when dyes are included. However, Cys–Cys distances from the two simulation sets (C) indicate that average IDR dimensions are essentially identical with and without dyes (see also Fig. 4B). Taken together, these results indicate that the dyes do not appreciably perturb the conformational ensemble of the IDRs; the small differences in transfer efficiencies likely arise from the dyes' excluded volume and slightly anisotropic positioning in the local protein environment (see Fig. S5). R denotes the Pearson correlation coefficient and CCC the concordance correlation coefficient.

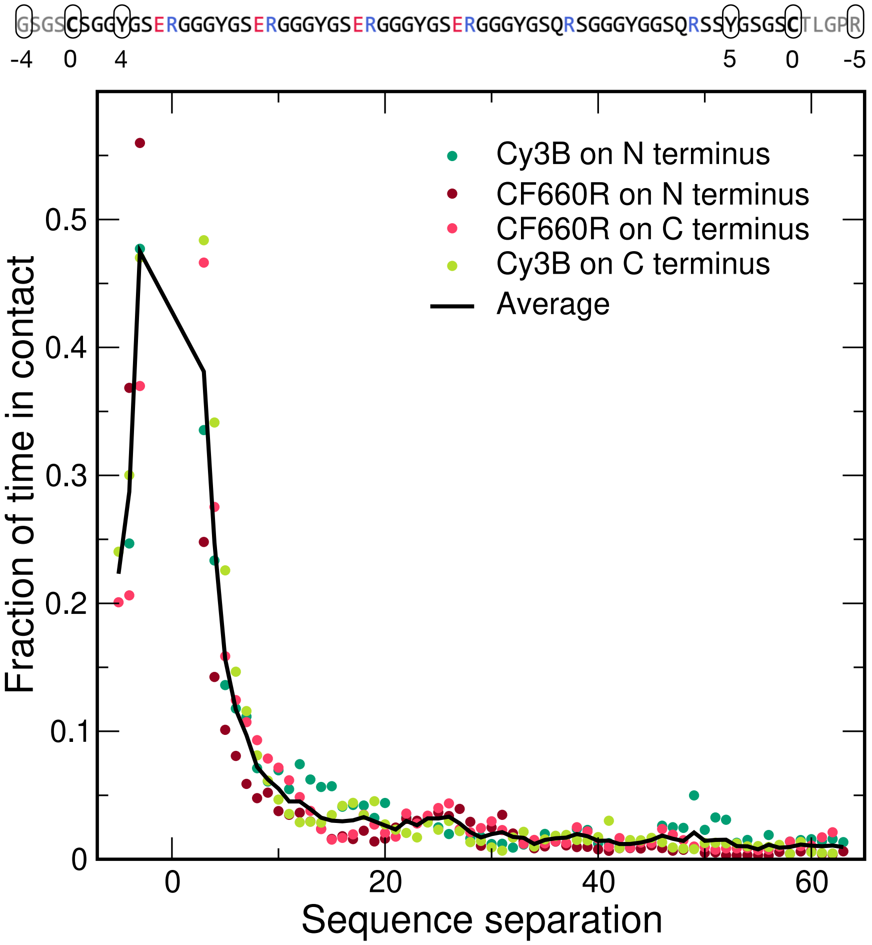

Figure S5: Fraction of time (normalized per simulation length) that the dyes are in contact with protein residues as a function of sequence separation. Negative sequence separation values indicate residues located closer to the termini than the Cys positions used for labeling (see schematic above the plot). The dyes predominantly contact neighboring residues, with slightly longer interaction times observed near the termini. This trend is consistent with the slightly reduced transfer efficiencies in simulations with dyes compared to those without dyes (Fig. 4 and Fig. S4). For details on the calculation of the fraction of time in contact, see Methods.

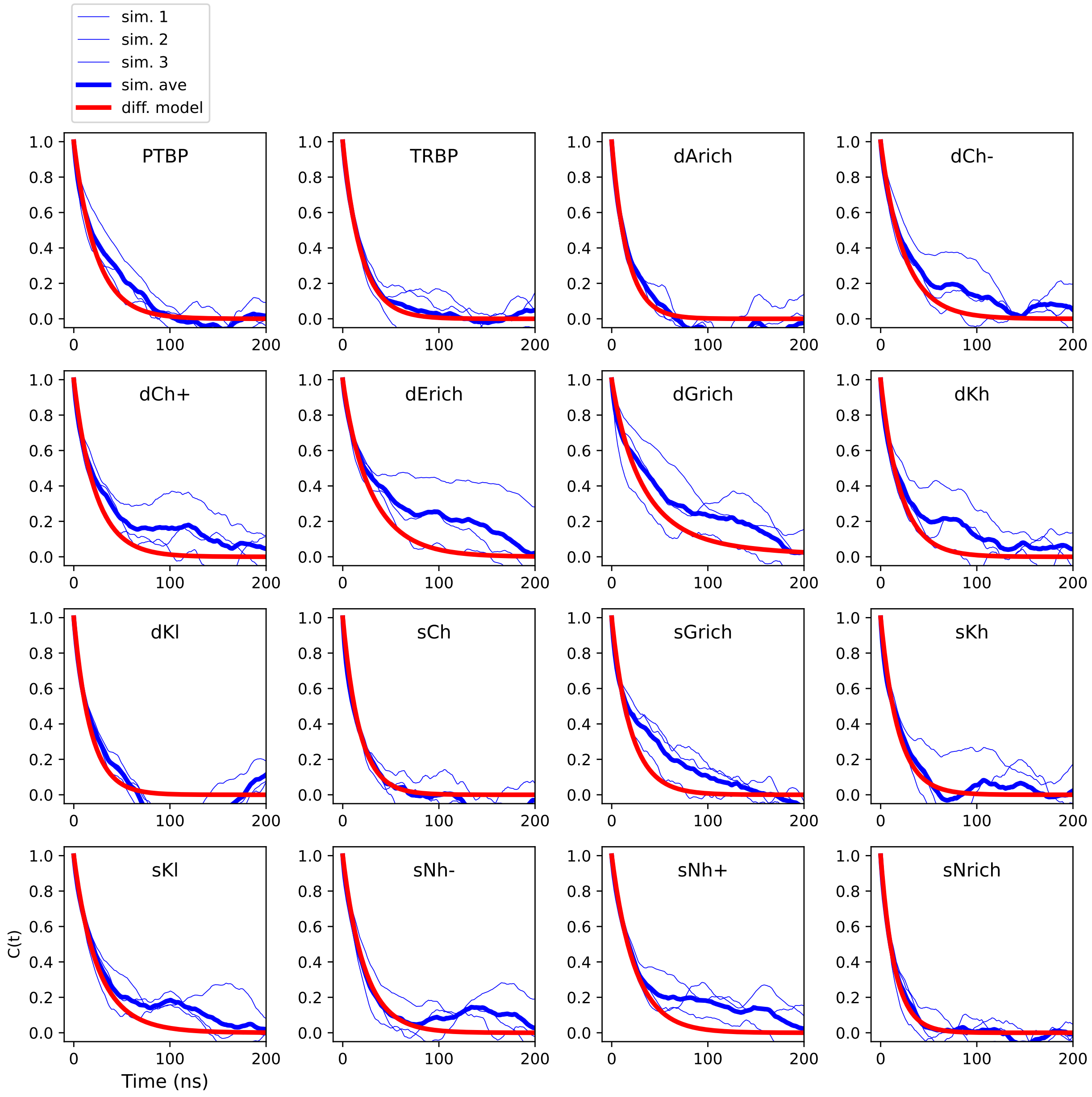

Figure S6: End-to-end distance correlation functions from the simulations. For each linker IDR, the correlation functions are plotted for each simulation replicate individually (thin blue lines), for the average over the three replicates (thick blue lines), and for the prediction using the one-dimensional diffusion model (thick red lines).

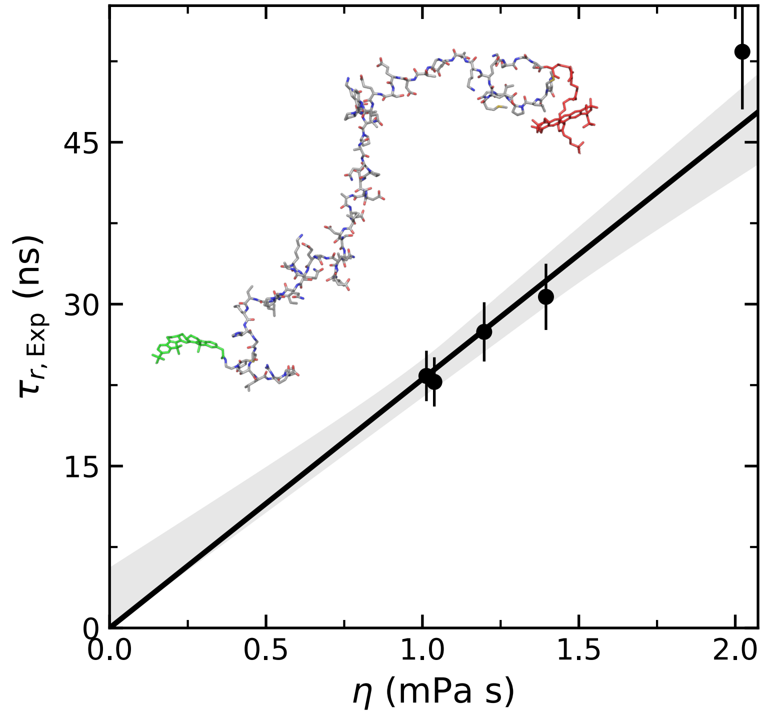

Figure S7: Quantifying internal friction in dCh− from solvent viscosity-dependent chain reconfiguration times. The internal friction time,[4] τ_i, results from extrapolating the measured reconfiguration times, τ_r, to zero solvent viscosity. Here the fit was constrained to yield $\tau_i \geq 0$, since internal friction times cannot take negative values. Inset: representative snapshot of dCh−.

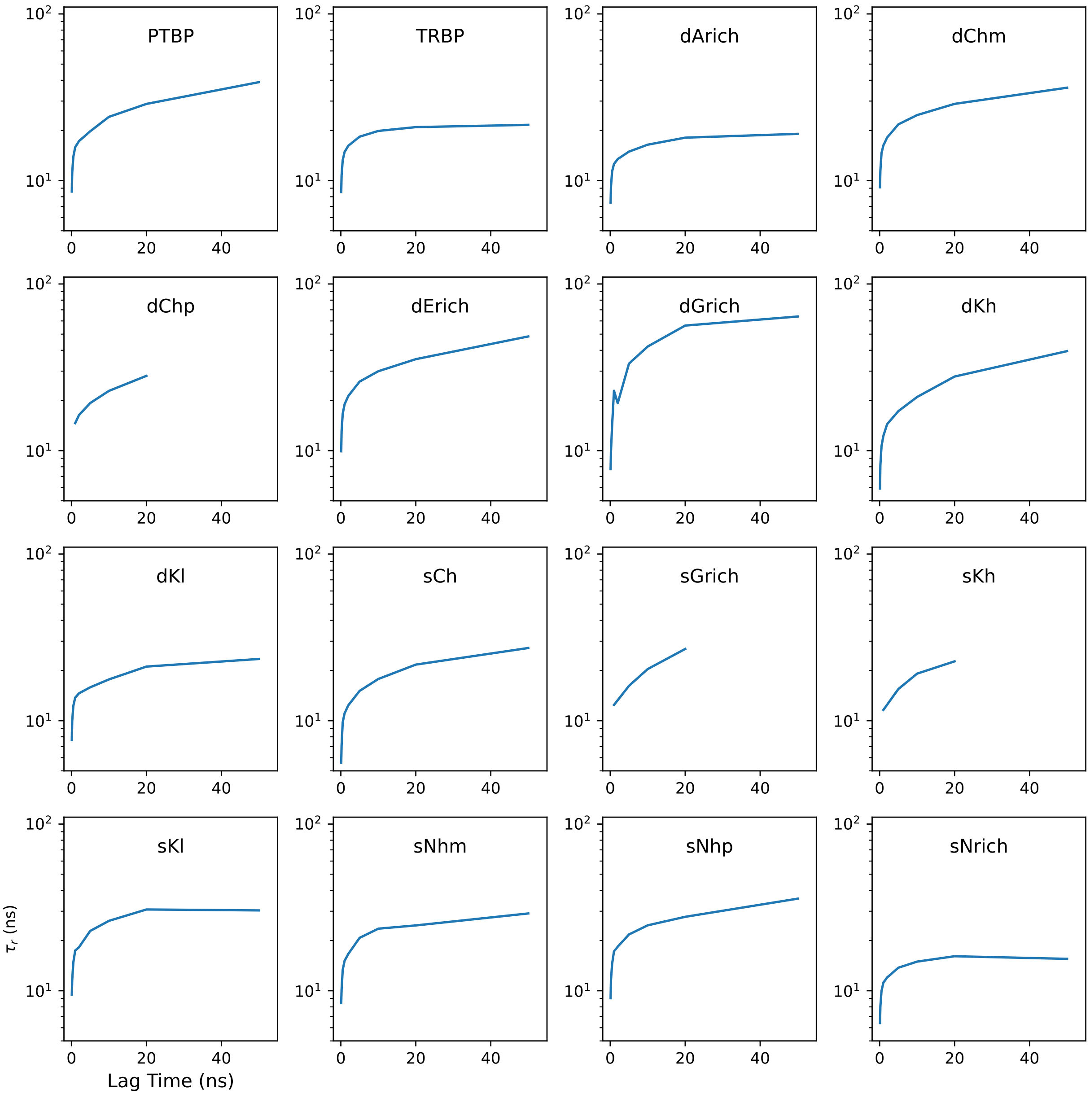

Figure S8: Dependence of reconfiguration time inferred from diffusion model on lag time (using data from single lag to fit model).

Supporting Tables

Table S1: Sequences of all linker IDRs used in simulations and experiments. Donor-/acceptor-labeling at the termini was site-selective.[1] R denotes the red dye (CFR660R, net charge $Q = -1$); G denotes the green dye (Cy3B, net charge $Q = 0$). Cys residues (positions 5 and 63) were used for labelling. 'GR' labelling refers to Cy3B at position 5 and CF660R at position 63, while 'RG' indicates the opposite.

Linker	Sequence	Site-specific labelling
sGrich	GSGSCSGGYGSERGGGYGSERGGGYGSERGGGYGSERGGGYGSQRSGGGYGGSQRSSYGSGSCTLGPR	GR
dκh	GSGSCAMGGGPGPGTDFTSDQADFPDTLFQEFEPPAPRPGLAGGRPGDAALLSAAYGRRRLLCTLGPR	GR
dGrich	GSGSCGPRTGLEGAGMAGGSGQQKRVFDGQSGPQDLGEAYRPLNHDGGDGGNRYSVIDRIQECTLGPR	GR
sPTBP	GSGSCRPDLPSGDSQPSLDQTMAAAFGLSVPNVHGALAPLAIPSAAAAAAAAGRIAIPGLAGCTLGPR	RG
sCh	GSGSCKKAQPEMNDKDDNESGNEDAEENHDDEEDENEEEDRQVDQASKNKESKRKAQNKREDCTLGPR	GR
sNrich	GSGSCLDQEDNNGPLLIKTANNLIQNNSNMLPLNALHNAPPMHLNEGGISNMRVNDSLPSNTCTLGPR	GR
sκh	GSGSCKPRRLSKLRRSKKPADEENNAASQDPTVEATQERGQASEDPENAANNAKQAKPTSDDCTLGPR	RG
dCh+	GSGSCQTPLKRIKVKTPGKSGAAAREGSVVSGTDGPTQTGKPERRKRLNPPKDKLIDMDDADCTLGPR	RG
dCh−	GSGSCMGLPTGMEEKEEGTDESEQKPVVQTPAQPDDSAEVDSAALDQAESAKQQGPILTKHGCTLGPR	RG
sκl	GSGSCAPEGVFKLPAPPKEIKKSEKSEKSSDSINDKKEVESTTKTAATTTTTKKGTDNNTQFCTLGPR	RG
sNh+	GSGSCPEEIETRKKDRKNRREMLKRTSALQPAAPKPTHKKPVPKRNVGAERKSTINEDLLPPCTLGPR	GR
dArich	GSGSCLPTGAEGRDSSKGEDSAEETEAKPAVVAPAPVVEAVSTPSAAFPSDATAEQGPILTKCTLGPR	RG
dErich	GSGSCEGEMFGVMSEENSVVLSVEQPAELKEVADVSPPPTTRNHTIEMKPPLSAQQSESNPVCTLGPR	RG
dTRBP	GSGSCLEPALEDSSSFSPLDSSLPEDIPVFTAAAAATPVPSVVLTRSPPMELQPPVSPQQSECTLGPR	RG
dκl	GSGSCLPTGMDQKTTELMKVDEPVSTQETLPPVIKMEPEPVPITETPTDENARQQGPILTKHCTLGPR	GR
sNh−	GSGSCKKKLQDTPSEPMEKDPAEPETVPDGETPEDENPTEEGADNSSAKMEEEEEEEEEEEECTLGPR	RG

Table S2: Reconfiguration times from nsFCS and FRET-corrected analyses. The reconfiguration times for each method are denoted τ_{nsFCS} and τ_{FRET}; their statistical uncertainties are fixed at 10% of the corresponding value (determined from repeat measurements; see Methods). The combined value τ_r is the arithmetic mean of τ_{nsFCS} and τ_{FRET}. The uncertainty $\Delta\tau_r$ is the quadratic combination of the statistical and systematic uncertainties from both methods.

Linker	τ_{nsFCS} (ns)	$\Delta\tau_{\mathrm{nsFCS,\,syst}}$ (ns)	τ_{FRET} (ns)	$\Delta\tau_{\mathrm{FRET,\,syst}}$ (ns)	$\boldsymbol{\tau_r \pm \Delta\tau_r}$ **(ns)**
sGrich	28	5.0	29	0.3	**29 ± 6**
dκh	37	8.0	46	0.0	**42 ± 10**
dGrich	30	2.7	32	0.5	**31 ± 5**
sPTBP	28	2.3	30	3.8	**29 ± 6**
sCh	26	1.6	30	0.7	**28 ± 4**
sNrich	25	1.7	27	0.2	**26 ± 4**
sκh	29	2.0	31	0.1	**30 ± 5**
dCh+	26	1.9	32	1.3	**28 ± 5**
dCh−	23	0.2	–	–	**23 ± 3**
sκl	23	0.7	25	0.8	**24 ± 4**
sNh+	27	2.1	32	4.3	**30 ± 6**
dArich	22	0.4	25	0.1	**24 ± 3**
dErich	25	1.2	30	0.4	**28 ± 4**
dTRBP	24	1.5	27	2.5	**26 ± 5**
dκl	21	1.5	–	–	**21 ± 3**
sNh−	30	1.5	36	1.2	**33 ± 5**

Table S3: Reweighting results. Columns report the initial and reweighted means of interdye distance r, FRET efficiency E, orientation factor κ^2, and the variance of E. Experimental targets are listed as E_{exp} and $\mathrm{Var}(E)_{\mathrm{exp}}$. Additional diagnostics include χ^2, the entropy change ΔS, the "temperature" parameter Θ, and the number of frames used N_{eff}.

Linker	r_{init} (nm)	r_{rew} (nm)	E_{init}	E_{rew}	E_{exp}	κ^2_{init}	κ^2_{rew}	$\mathrm{Var}(E)_{\mathrm{init}}$	$\mathrm{Var}(E)_{\mathrm{rew}}$	$\mathrm{Var}(E)_{\mathrm{exp}}$	χ^2	ΔS	Θ	N_{eff}
sGrich	3.55	3.55	0.90	0.90	0.87	0.63	0.63	0.03	0.03	0.03	0.81	0.00	1.17e6	1.00
dκh	4.10	4.33	0.84	0.80	0.77	0.67	0.67	0.05	0.06	0.06	1.00	0.01	49.16	0.99
dGrich	4.07	4.41	0.82	0.76	0.73	0.63	0.64	0.06	0.08	0.08	1.00	0.02	41.91	0.98
sPTBP	5.09	5.09	0.67	0.67	0.66	0.67	0.67	0.10	0.10	0.09	0.27	0.00	1.03e20	1.00
sCh	4.24	5.11	0.82	0.67	0.64	0.67	0.68	0.05	0.09	0.10	1.13	0.18	16.52	0.84
sNrich	5.68	5.68	0.58	0.58	0.60	0.66	0.66	0.09	0.09	0.10	1.15	0.00	1.26e6	1.00
sκh	4.36	5.27	0.79	0.63	0.60	0.65	0.67	0.05	0.09	0.10	1.75	0.24	13.94	0.78
dCh+	4.57	5.34	0.75	0.62	0.59	0.66	0.67	0.07	0.10	0.11	1.35	0.11	23.41	0.90
dCh−	6.10	6.10	0.52	0.52	0.50	0.69	0.69	0.11	0.11	0.13	3.03	0.00	0.00	1.00
sκl	5.64	6.06	0.59	0.52	0.49	0.64	0.65	0.11	0.12	0.13	1.41	0.02	54.87	0.98
sNh+	5.59	5.95	0.60	0.54	0.51	0.66	0.66	0.11	0.11	0.13	1.64	0.02	64.85	0.98
dArich	6.05	6.24	0.52	0.49	0.47	0.65	0.65	0.10	0.11	0.13	1.30	0.01	47.88	0.99
dErich	5.51	6.05	0.60	0.51	0.48	0.65	0.65	0.11	0.12	0.13	1.48	0.04	40.49	0.97
dTRBP	6.30	6.41	0.49	0.47	0.46	0.66	0.66	0.11	0.11	0.13	1.12	0.00	49.48	1.00
dκl	6.60	6.57	0.43	0.44	0.44	0.64	0.64	0.10	0.12	0.14	1.02	0.02	20.51	0.98
sNh−	6.42	6.77	0.47	0.43	0.42	0.67	0.67	0.10	0.12	0.14	1.11	0.06	12.86	0.95

References

(1) Holla, A.; Martin, E. W.; Dannenhoffer-Lafage, T.; Ruff, K. M.; Konig, S. L.; Nuesch, M. F.; Chowdhury, A.; Louis, J. M.; Soranno, A.; Nettels, D.; others Identifying Sequence Effects on Chain Dimensions of Disordered Proteins by Integrating Experiments and Simulations. *JACS Au* **2024**, *4*, 4729–4743.

(2) Das, R. K.; Pappu, R. V. Conformations of intrinsically disordered proteins are influenced by linear sequence distributions of oppositely charged residues. *Proc. Natl. Acad. Sci. U.S.A.* **2013**, *110*, 13392–13397.

(3) Sawle, L.; Ghosh, K. A theoretical method to compute sequence dependent configurational properties in charged polymers and proteins. *J. Chem. Phys.* **2015**, *143*.

(4) Soranno, A.; Buchli, B.; Nettels, D.; Cheng, R. R.; Müller-Späth, S.; Pfeil, S. H.; Hoffmann, A.; Lipman, E. A.; Makarov, D. E.; Schuler, B. Quantifying internal friction in unfolded and intrinsically disordered proteins with single-molecule spectroscopy. *Proc. Natl. Acad. Sci. U.S.A.* **2012**, *109*, 17800–17806.
